# Life-threatening events in patients with LQT3 on and off beta-blockers: Insights from a case series

**DOI:** 10.1016/j.hroo.2025.11.005

**Published:** 2025-11-13

**Authors:** Alexis Hermida, Jean-Baptiste Gourraud, Isabelle Denjoy, Adrien Bloch, Vincent Probst, Fabrice Extramiana

**Affiliations:** 1 Cardiology, Arrhythmia, and Cardiac Stimulation Service, Amiens-Picardie University Hospital, Amiens, France; 2 EA4666 HEMATIM, University of Picardie-Jules Verne, Amiens, France; 3 L’institut du thorax, CNMR Maladies rythmiques héréditaires ou Rares, Service de Cardiologie et unité INSERM 1087, Centre hospitalier universitaire de Nantes, Nantes, France; 4 CNMR Maladies Cardiaques Héréditaires Rares, APHP, Hôpital Bichat, Paris, France; 5 Service de Biochimie Métabolique, Groupe Hospitalier Pitié-Salpêtrière, APHP, Paris, France; 6 Université Paris Cité, Paris, France

**Keywords:** Type 3 long QT syndrome, *SCN5A*, Life-threatening events, Implantable cardioverter-defibrillator, Beta-blockers

**Key Findings**

▪ Patients presenting with arrhythmic events at birth had a very poor prognosis.
▪ Arguments used by physicians to support the decision to implant an implantable cardioverter-defibrillator for primary prevention effectively identified patients who subsequently benefited.
▪ Data do not clearly link genetics to life-threatening event occurrence.

The efficacy of beta-blockers in type 3 long QT syndrome (LQT3) remains uncertain, with conflicting results in the literature.^1^^,^^2^ Wilde et al^1^ reported benefit in women, but this was not confirmed in a French cohort.^2^ This discrepancy has raised concerns that *confounding by indication*—that is, the preferential prescription of beta-blockers to the most severely affected patients—might partly explain the apparent lack of benefit observed in some studies. To explore this hypothesis, we conducted a detailed evaluation of patients with LQT3 who experienced life-threatening events (LTEs), either on or off beta-blocker therapy.

We included all patients with LQT3 from the Nantes and Paris reference centers (those in our previous publication,^2^ plus patients with LTEs during the first year of life). The anonymized database was registered with the French National Data Protection Commission (PI2022_843_0059). Documented sustained ventricular arrhythmia, torsades de pointes, aborted cardiac arrest (ACA), sudden cardiac death, and appropriate implantable cardioverter-defibrillator (ICD) shocks were considered LTEs.

Our study included 148 patients from 55 families, each carrying 1 of 23 pathogenic/probably pathogenic *SCN5A* variants (22 missense, 1 deletion). 19 patients (13%) experienced LTEs (12 off beta-blockers, 7 on treatment).

Among the 12 patients with LTE off beta-blockers (Figure 1A):

• 6 had LTE as the index event (3 ACA, 1 ventricular fibrillation, 2 sustained ventricular arrhythmia). 4 received ICDs but had no LTE recurrence off beta-blockers during long-term follow-up.
• The other 6 (diagnosed after syncope in 1, fortuitously in 3, and via cascade screening in 2) had prophylactic ICDs. 2 received flecainide [1 woman carrying the p.(Glu1784Lys) variant and 1 carrying the p.(Asp1790Gly) variant]. All experienced appropriate ICD shocks during follow-up.

**Figure 1:**
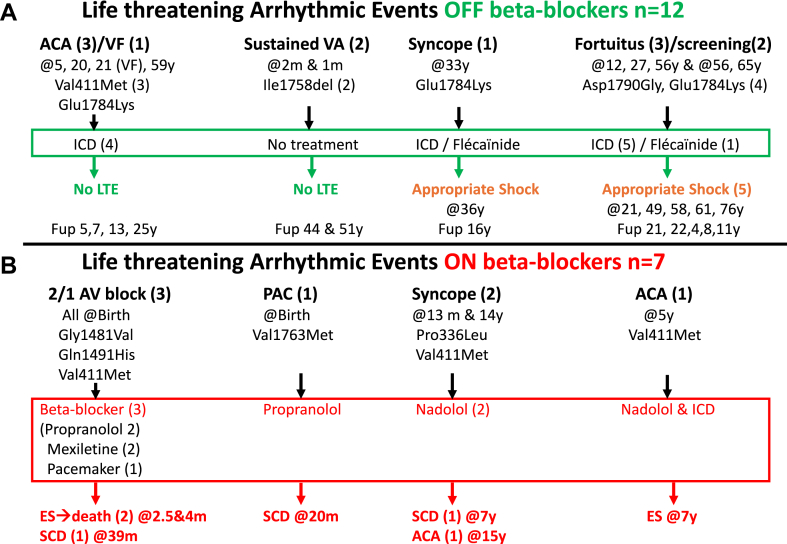
Patients with LTE off (**A**) and on (**B**) beta-blockers. ACA = aborted cardiac arrest; AV = atrioventricular; ES = electrical storm; Fup = follow-up; ICD = implantable cardioverter-defibrillator; LTE = life-threatening event; PAC = premature atrial contraction; SCD = sudden cardiac death; VA = ventricular arrhythmia; VF = ventricular fibrillation.

8 patients (67%) were index cases; 7 (58%) were males. The median age at diagnosis was 24 (6.7–56) and at first event 28.5 years (8.7–58.7). The mean corrected QT interval was 514 ± 55 ms. Variants were in the pore (n = 5; 42%) and in the C-terminus (n = 7; 58%).

7 patients, all probands, had LTE on beta-blockers (Figure 1B).

• 4 were diagnosed at birth (3 with 2:1 atrioventricular block and 1 with premature atrial contractions detected on auscultation, which prompted electrocardiographic recording), all treated with propranolol. 2 died during electrical storms (at 2.5 and 4 months of age) despite a treatment attempt with mexiletine, and 2 died suddenly at 20 and 39 months.
• 3 others were diagnosed after the age of 1 year. 1 had an ACA at 5 years off treatment, was then implanted with an ICD, and prescribed nadolol, but later had an electrical storm. The other 2, treated with nadolol after syncope, also had severe outcomes (1 died suddenly at 7 years, the other had an ACA at 15 years).

All 7 with LTE on treatment were index cases; 5 (71%) were male. The median age at diagnosis was 0 (0–5) and at event 3 years (0–7). The mean corrected QT interval was 580 ± 129 ms. 4 *SCN5A* variants (57%) were located in the pore; none were in the C-terminus.

Patients with LTE on beta-blockers had earlier events (3 [0–7.0] vs 28 [9–59] years; *P* = .003) and more often carried variants in interdomain linkers/transmembrane regions (n = 7 [100%] vs n = 5 [42%]; *P* = .02).

Our data suggest the following patterns:

First, as previously described,^3^ patients presenting with arrhythmic events at birth had a very poor prognosis (all 4 died). In these cases, beta-blockers did not seem protective.

Second, 5 patients diagnosed after an ACA (youngest at 5 years) received ICDs. 1, on nadolol, had an electrical storm later. The 4 not treated with beta-blockers had no LTE during a 5–25 years’ follow-up. These limited data do not prove a protective effect of nadolol but do not exclude it.

Third, 6 patients experienced LTEs (appropriate ICD shocks) after prophylactic ICD implantation (none on beta-blockers) during a 4–22 years’ follow-up. 4 had no history of syncope, yet, among 8 patients implanted for the same indication in the entire cohort,^2^ this corresponded to a 50% rate of appropriate shocks. Thus, the arguments used by physicians to support the decision to implant an ICD for primary prevention effectively identified patients who subsequently benefited.

Fourth, genetically, 6 patients carried the p.(Val411Met) variant, known as particularly severe,^3^ whereas 6 carried the p.(Glu1784Lys), previously described as benign.^1^ More than half of patients with LTEs off beta-blockers had C-terminal variants—absent in patients with LTEs on beta-blockers. However, this association is very weak when considering the small size of the cohort, and data do not clearly link genetics to LTE occurrence.

In conclusion, this focused analysis of patients with LQT3 experiencing LTEs—not previously described in our national cohort—highlights the difficulty of identifying those who benefit from beta-blockers. Monotherapy may be insufficient for severe phenotypes, especially in infancy. Nevertheless, ICDs are associated with complications and should be considered carefully. Sodium channel blockers may help, although data in LQT3 remain limited. Larger collaborative studies are needed to clarify LQT3 natural history and optimize treatment.

## Disclosures

The authors have no conflicts to disclose.
